# Eleven tenets for the integrated, evolving and sustainable management of European Research Infrastructure Consortia

**DOI:** 10.3897/BDJ.14.e212181

**Published:** 2026-10-01

**Authors:** Christos Arvanitidis, Cristina Isabel Huertas Olivares, Mike Elliott, Alberto Basset, Anne Fouilloux, Ohad Graber-Soudry, Dimitris Koureas, Lucas de Moncuit, Roland Roberts, Antonio José Saenz-Albanes, Peter van Tienderen

**Affiliations:** 1 LifeWatch ERIC, Seville, Spain LifeWatch ERIC Seville Spain; 2 Institute of Marine Biology, Biotechnology and Aquaculture (IMBBC), Hellenic Centre for Marine Research (HCMR), Former US Base at Gournes, Crete, Greece Institute of Marine Biology, Biotechnology and Aquaculture (IMBBC), Hellenic Centre for Marine Research (HCMR) Former US Base at Gournes, Crete Greece; 3 International Estuarine & Coastal Specialists (IECS) Ltd, Leven, United Kingdom International Estuarine & Coastal Specialists (IECS) Ltd Leven United Kingdom; 4 School of Environmental & Life Sciences, University of Hull, Hull, United Kingdom School of Environmental & Life Sciences, University of Hull Hull United Kingdom; 5 Università del Salento, Department of Biological and Environmental Sciences and Technologies (DiSTeBA), Centro Ecotekne, Lecce, Italy Università del Salento, Department of Biological and Environmental Sciences and Technologies (DiSTeBA) Centro Ecotekne, Lecce Italy; 6 LifeWatch ERIC, Lecce, Italy LifeWatch ERIC Lecce Italy; 7 X-officio, Lund, Sweden X-officio Lund Sweden; 8 Naturalis Biodiversity Center, Leiden, Netherlands Naturalis Biodiversity Center Leiden Netherlands; 9 Retired, National Science Foundation (NSF), Alexandria, VA, United States of America Retired, National Science Foundation (NSF) Alexandria, VA United States of America; 10 LifeWatch ERIC, Amsterdam, Netherlands LifeWatch ERIC Amsterdam Netherlands

**Keywords:** adaptive management, eleven-tenet framework, European Research Infrastructure Consortia, scientific excellence, sustainable governance

## Abstract

Research Infrastructures are increasingly central to scientific discovery, innovation and evidence-informed policy, but their management requires the integration of many dimensions that are often treated separately. This paper proposes an eleven-tenet framework for the integrated, evolving and sustainable management of European Research Infrastructure Consortia, extending existing tenet-based approaches by adding the explicit dimension of enabling scientific excellence. The framework brings together scientific, technological, economic, administrative, political, legal, societal, environmental, ethical, cultural and communication considerations into a coherent structure. It is intended to support strategic reflection, gap analysis, adaptive management and future assessment of ERICs, while recognising that the challenges addressed are relevant beyond the European RI and ERIC context.

## Abbreviations

- CARE: Collective Benefit, Authority to Control, Responsibility and Ethics.
- DMP(s): Data Management Plan(s).
- FAIR: Findable, Accessible, Interoperable and Reusable.
- EOSC: European Open Science Cloud.
- ERA: European Research Area.
- ERIC(s): European Research Infrastructure Consortium(a).
- ESFRI: European Strategy Forum on Research Infrastructures.
- FOREST: Financial and Organiszational Sustainability, Openness, Representative Governance, Equity, Accessibility and Anti-Oppression, Sharing of Knowledge and Transparency.
- K-M GBF: Kunming-Montreal Global Biodiversity Framework.
- KPI(s): Key Performance Indicators.
- NSF: National Science Foundation (USA).
- PEST: Political, Economic, Social, Technological.
- PESTLE: Political, Economic, Social, Technological, Legal, Environmental.
- POSI: Principles of Open Scholarly Infrastructure.
- RI(s): Research Infrastructure(s).
- RoI: Return on Investment.
- SES: Social-Ecological System.
- SMART: Specific, Measurable, Achievable, Relevant, Time-bound.
- SWOT: Strengths, Weaknesses, Opportunities, Threats.

## Introduction

Research Infrastructures are complex socio-technical systems that play a central role in advancing scientific knowledge, fostering innovation and supporting evidence-based policy. Their effective management is therefore critical not only for ensuring scientific progress, but also for delivering broader societal, economic and environmental benefits. However, the increasing scale, diversity and interconnectedness of RIs within the ERA have made their management inherently complex, requiring coordinated approaches that integrate scientific, technological, organisational and societal dimensions.

Within the European Union, substantial investments have been made in RIs, which are defined as facilities, resources and services used by research communities to conduct research and promote innovation (e.g. European Strategy Forum on Research Infrastructures (ESFRI) (2020), European Strategy Forum on Research Infrastructures (ESFRI) 2021). Amongst these, ERICs constitute legally established entities operating under a common regulatory framework. While the ERIC Regulation (Council of the European Union 2009) provides the legal basis for their establishment, operation and dissolution, their governance structures and operational modalities are defined through individual Statutes (ERIC Practical Guidelines 2025). As such, ERICs represent the primary legally constituted infrastructures operating across Europe.

Although this study focuses on ERICs, comparable large-scale research infrastructure arrangements exist internationally, including the United States NSF major facilities and mid-scale research infrastructure, Australia’s National Collaborative Research Infrastructure Strategy and China’s major science and technology infrastructures, indicating that the need for integrated, sustainable and adaptive RI management extends well beyond the ERA.

To promote interdisciplinary and cross-domain collaboration, the European Commission has organised RIs into thematic Science Clusters, which are further embedded within the evolving EOSC ecosystem. However, these Clusters do not possess legal status and operate primarily as coordination mechanisms. Consequently, the responsibility for ensuring integrated, adaptive and sustainable operations rests largely with the individual ERICs, reinforcing the importance of robust and coherent management frameworks.

The ESFRI has developed a comprehensive framework for the mandatory monitoring of the performance of Landmark RIs (fully implemented, research facility (e.g. ERIC) recogniszed by ESFRI), structured around three main components or cases: (a) general information, addressing political support, financial commitment and inclusion in national roadmaps; (b) the scientific case, evaluating scientific excellence and impact; and (c) the implementation case, assessing governance, operations and service delivery (European Strategy Forum on Research Infrastructures (ESFRI) 2024). Excluding the general information component, this framework spans a wide range of thematic areas, covering scientific, organisational and operational aspects of ERIC performance. While highly detailed and operational, this monitoring approach does not explicitly provide a unifying conceptual structure linking these dimensions. In the context of ERICs, the objective is not only to guarantee effective operation, but to enable sustainable and impactful delivery of scientific and societal value. This requires, first, clearly defining the challenges to be addressed and then applying a proposed ‘eleven-tenet framework’ in a systematic and rigorous manner to ensure that all relevant dimensions are considered (see below, Table 1). In this sense, the tenets should be used as a structured checklist to identify potential gaps or impediments that may hinder the successful functioning and long-term sustainability of the infrastructure. By explicitly addressing each of the proposed eleven dimensions, the framework supports a comprehensive and balanced approach to managing ERICs, ensuring that no critical aspect is overlooked.

Previous policy-orientated work has also highlighted the need for general principles to guide the quality, access, impact and sustainability of RIs. For example, the League of European Research Universities proposed four “golden principles” for RIs, emphasising adherence to access principles and FAIR data policies, transparent and up-to-date information on services and access conditions, stable organisational embedding and clear indicators of operational and scientific quality (Väänänen and van Tienderen 2017). This approach is particularly relevant because it links access, openness, organisational accountability and performance evidence and further recommends the development of a standard checklist to assess whether ERICs adhere to these principles. While this provides an important operational reference point, the present study seeks to broaden the conceptual basis for ERIC management by integrating scientific, technological, economic, administrative, political, legal, societal, environmental, ethical, cultural and communication dimensions into a single eleven-tenet framework.

Recent work on open access research infrastructures (Spinello et al. 2021) also reaches a similar conclusion from a different perspective. Haak et al. (2025), focusing on ‘open by design’ approaches, argue that existing open-science frameworks, including FAIR, CARE, POSI and FOREST, provide important principles and evaluative lenses, but do not by themselves offer an integrated design and implementation framework for infrastructure builders. Their approach is particularly relevant here because it similarly emphasises the need to move from fragmented checklists towards a coherent framework through which openness, governance, sustainability, community engagement, interoperability and evaluation can be embedded into the lifecycle of RIs.

Similar multidimensional approaches have been developed in other domains. In business and organisational analysis, the PEST framework (political, economic, social and technological) and its subsequent extensions (PESTLE; adding legal and environmental dimensions) have been widely used to capture the complexity of organisational environments. In the specific context of RI strategy, Marafioti et al. (2026) also underline the relevance of PESTLE analysis as a macro-environmental tool for understanding the political, economic, sociological, technological, legal and environmental factors shaping RI operations, particularly in transnational infrastructures where trends may need to be assessed at both national-node and European levels. In environmental management, particularly in marine ecosystems, these approaches have been further expanded to six, then seven, then ten tenets, which were developed separately from PESTLE to include additional dimensions such as, ethical, cultural and communication aspects, leading to the formulation of the ten-tenet framework for integrated and sustainable management (Elliott 2013, Barnard and Elliott 2015, Elliott et al. 2025).

Striking parallels can be identified between these ten-tenets and the thematic areas covered by the ESFRI monitoring framework. The eleven-tenet framework proposed here addresses this gap by offering an integrated management structure through which ESFRI monitoring dimensions can be interpreted, aligned and implemented. However, an important distinction must be made: in the context of ERICs, scientific excellence is not an intrinsic property of the infrastructure itself, but rather an outcome generated by the research communities it supports. The role of the infrastructure is, therefore, to enable such excellence through the provision of high-quality data, services and collaborative environments, which, in turn, must be amongst the parameters on which ERICs are assessed, as proxies for scientific excellence.

Building on this distinction, this study extends the original ten-tenet framework by introducing an additional tenet, enabling scientifical excellence, thereby forming a set of *eleven-tenets* (Table 1), tailored to the specific context of ERICs.

The ERICs present a number of inherent strengths, including their capacity to integrate large and diverse datasets, support interdisciplinary collaboration and provide shared services to wide user communities. However, they also face significant challenges, such as fragmentation across domains, complexity in governance and coordination, difficulties in ensuring long-term financial sustainability and the need to continuously adapt to evolving scientific, technological and policy landscapes. These challenges often result in gaps between data generation and effective use, as well as between scientific potential and societal impact. In this context, the application of the tenet-based framework provides a systematic and rigorous approach to both enhancing the inherent strengths of ERICs and addressing their limitations. By ensuring that all critical dimensions are considered in an integrated manner, the framework supports the identification and removal of impediments to effective operation, thereby improving the capacity of ERICs to deliver sustainable, impactful and policy-relevant outcomes.

The interdependence of the eleven-tenets and their organisation into core, structural and contextual tiers can be conceptualised through a concentric model (Fig. 1), which illustrates their integrated and non-hierarchical nature. The three tiers represent complementary levels of functionality within the framework. The **core tier** defines the fundamental purpose and enabling conditions of the ERIC, focusing on its capacity to deliver scientific value through the integration of scientific excellence, technological capability and financial sustainability. The **structural tier** encompasses the governance and organisational mechanisms required to ensure that the infrastructure operates effectively within its institutional and policy context, including administrative, legal and political alignment. The **contextual tier** addresses the broader societal, environmental and cultural environment in which the infrastructure operates, ensuring its relevance, legitimacy and long-term sustainability through responsiveness to societal needs, ethical considerations and effective communication. Together, these tiers illustrate the multi-layered nature of RI management, where purpose, structure and context must be coherently aligned.

The aim is to assess whether this extended framework can provide a coherent and comprehensive basis for supporting the integrated, adaptive and sustainable management of ERICs, while remaining fully aligned with the ESFRI monitoring approach. While highly detailed and operational, the ESFRI monitoring framework is primarily designed to assess the performance of ERICs across a range of dimensions, rather than to provide a unifying conceptual structure for their management. As such, it offers a comprehensive set of criteria and indicators, but does not explicitly integrate these into a coherent framework that links the different dimensions of ERIC functioning. This creates a gap between performance monitoring and the systematic management of ERICs as complex socio-technical systems. The eleven-tenet framework proposed here addresses this gap by providing an integrated structure through which these dimensions can be understood, aligned and implemented, thereby supporting more coherent and effective management practices.

## What do the eleven-tenets refer to?

Building on the framework presented above, the eleven tenets are described below according to their organisation into core, structural and contextual tiers.

### A. Core tier

**A1. Enabling scientific excellence**

The aim of this tenet is to ensure that the ERIC provides the conditions necessary for research communities to achieve scientific excellence. In the context of ERICs, scientific excellence is not an intrinsic property of the infrastructure itself, but an exogenous outcome generated by its user communities. The role of any ERIC is, therefore, to enable such excellence through the provision of high-quality FAIR-compliant data, advanced reproducible analytics, robust computational environments and effective support to user communities. In this context, while all tenets are expected to be implemented in a scientifically robust manner, scientific excellence enabling is conceptually distinct, as it defines the primary purpose of ERICs, namely to enable high-quality scientific outcomes delivered by their user communities.

In line with the ESFRI monitoring framework, evidence for this tenet should include the quality, accessibility and interoperability of services and data; the strength and diversity of user communities; the effectiveness of access policies (including transnational access); and the degree to which the infrastructure supports high-impact scientific outputs. This may be demonstrated through indicators, such as user uptake, research products enabled by the infrastructure (e.g. datasets, workflows, publications), contributions to interdisciplinary research and engagement in co-design and co-creation processes with scientific communities.

Furthermore, this tenet requires the ERIC to continuously evolve its services in response to scientific needs, emerging methodologies and technological developments, ensuring that it remains a relevant and effective enabler of excellence across domains and disciplines.

**A2. Technological feasibility**

The objective of this tenet is to ensure that the ERIC possesses the necessary technological capacity to deliver its mission effectively, currently and in the longer term. This includes the availability, performance and scalability of infrastructure, services and digital systems. The technological feasibility also requires digital operational readiness, including a coherent service architecture, interoperable APIs, data and metadata pipelines, authentication and authorisation mechanisms, cybersecurity controls, monitoring dashboards, backup and disaster-recovery procedures, user support mechanisms, documentation and clearly defined service-level expectations. The ESFRI monitoring process emphasises the need to demonstrate: (a) interoperability with other RIs (e.g., Smith et al. (2022)), especially those within the same Science Cluster, as well as with the EOSC landscape; (b) access policy and, where possible, funding support for access; (c) data management strategies, including DMPs and alignment with FAIR principles; (d) use of national, European and international repositories; (e) capability to federate and integrate technical and community components, by connecting distributed open data resources, reproducible analytics, services and mobilised communities into an interoperable research ecosystem; (f) ability to integrate emerging technologies; (g) management of infrastructure for the entire lifecycle, upgrade strategies and the adequacy of technical support and expertise. Technological feasibility is, therefore, well captured by the ESFRI monitoring framework, which requires ERICs to demonstrate interoperability, FAIR data management, access arrangements, repository integration, technological adaptability and lifecycle planning.

**A3. Economic viability**

This tenet addresses the long-term financial sustainability of the ERIC, ensuring that both its strategic planning, implementation and operations are adequately and sustainably supported. The ESFRI guidelines require evidence of secured member contributions (EU Member States may be the only members in some ERICs) and financial commitments over multi-annual periods. Robust financial governance mechanisms (i.e., auditing of accounting and budget systems) is a prerequisite for the successful operation of an ERIC. In cases in which decommissioning is foreseen, the relevant costs should be estimated, *a priori*. In addition, the ERIC should demonstrate diversification of income streams, including participation in competitive funding programmes, cost recovery schemes for the provision of their services, collaboration with the industry and provide forward-looking financial planning and cost-efficiency analysis to ensure resilience under changing funding conditions. A SWOT analysis forms part of this tenet. In addition, a risk strategy for the estimation of potential risks is an essential part of this tenet; it includes: (a) assessing the causes and/or drivers of risks and their potential consequences; (b) evaluating the likelihood and expected impact of risks, including their possible effects on costs, revenues and economic viability; (c) defining prevention and mitigation measures to avoid risk materialisation and reduce the severity of consequences; and (d) using tools such as risk registries and bow-tie analysis, to support implementation of the strategy.

### B. Structural tier

B1. Administrative achievement (*)

This tenet ensures that the ERIC operates effectively and efficiently as an autonomous organisation. This includes clearly defined governance arrangements, decision-making processes and internal coordination mechanisms. The ESFRI monitoring requires evidence of management effectiveness, risk management practices (see tenet A3 above), internal audit mechanisms and the use of performance indicators (e.g., KPIs) linked to strategic objectives. Regular reporting, evaluation cycles and the capacity to adapt organisational processes over time are also key elements of this tenet. Administrative achievement may also be supported by operational dashboards, service monitoring, usage analytics, access statistics and FAIRness indicators. Relevant evidence may include administrative service availability, incident frequency, recovery time, active users, supported datasets and workflows, API usage, support response time, security reviews and user satisfaction.

B2. Political alignment (*)

This tenet focuses on the ERIC’s strategic direction, that needs to be aligned with prevailing political and policy research priorities, at all possible levels: European, national, regional and local. This vertical alignment should be complemented by horizontal integration with similar non-ERIC bodies, initiatives and infrastructures, so that the ERIC is positioned not in isolation, but within a broader ecosystem of related organisations and policy-relevant actors. To this end, it is very important to collect relevant data and develop narratives in order to provide adequate information on the RoI to the partnering countries. The ESFRI monitoring emphasises the need to demonstrate strong political support from members of the ERIC, for example, with their inclusion in national roadmaps and sustained commitment over time. In addition, the ERIC should provide evidence of alignment with European and global policy frameworks and approaches, such as the K-M GBF and One Health and demonstrate its contribution to policy-relevant outcomes, including participation in policy dialogues and advisory processes. Therefore, a mapping of the research products against political and policy priorities is an essential step in order for a RI to demonstrate its contribution to all of the above needs.

B3. Legal governance (*)

This tenet ensures that the ERIC operates fully within the applicable legal frameworks at national, European and international levels. For example, the ESFRI guidelines require evidence of compliance with the ERIC Regulation, as well as national legal frameworks in participating countries. This includes the existence of statutes, internal rules, policies and strategies, as well as contractual arrangements, governing operations, relevant data and services. Particular attention is given to issues such as intellectual property rights, data protection and liability management, ensuring that legal risks are identified and mitigated. In digital ERICs, legal governance also depends on robust digital governance mechanisms, including clear data licensing, controlled access where required, auditability of data and service use, identity and access management, cybersecurity-by-design, vulnerability management, backup and recovery procedures, documented incident response processes and compliance with relevant procurement, environmental and energy-related obligations.

(*) These tenets are encompassed within governance, understood as the combined framework of policies, politics, administration and legislation.

### C. Contextual tier

**C1. Societal desirability**

The objective of this tenet is to ensure that the ERIC remains relevant and responsive to societal needs. It requires the regular collection of the needs and the active engagement of users and stakeholders in the design and development of services, preferably through structured co-design and co-creation processes. In addition, it responds to the European, national and regional relevant research priorities and offers assistance for the implementation of the relevant policies (see also tenet B2 above). The guidelines of ESFRI further require evidence of user access policies, transnational access provision and the extent to which services address societal challenges and policy needs. Performance indicators may include user uptake, diversity of user communities and documented impacts on science, society and policy.

**C2. Environmental / energy / ecological sustainability**

The aim of this tenet is to address the need of the ERIC to operate with minimal environmental and energy impact, while actively contributing to the sustainability of ecological systems, the last feature being necessary especially for the ERICs more related to the environment domain, that is those forming the ENVRI Science Cluster (https://envri.eu/). Beyond compliance with relevant legislation (e.g., The European Parliament and the Council of the European Union (2023); Commission (2026); see also tenet B2), the ERIC is expected to demonstrate measurable performance through data, indicators and documented trends over time. In line with ESFRI monitoring guidelines, this includes evidence on energy consumption, carbon footprint, resource efficiency and mitigation measures, as well as the integration of environmental considerations into the infrastructure planning, development and operations. The development and implementation of a comprehensive environmental and energy policy, with clearly defined targets and monitoring mechanisms, is therefore deemed as essential. Sustainable operation also requires responsible digital practices, including energy-aware computing, efficient storage policies, rational data-retention strategies, optimisation of cloud and computing resources, monitoring of the digital carbon footprint, reuse of shared services and avoidance of unnecessary duplication of digital infrastructure.

**C3. Cultural inclusiveness**

This tenet serves the necessity of the ERIC to operate in an inclusive and culturally sensitive manner. The ESFRI-related expectations include the promotion of diversity, gender equality and equal opportunities within governance and operations, in line with the United Nations Disability Inclusion Strategy (Chief Executives Board for Coordination 2026) and its policy of inclusiveness. Evidence should demonstrate inclusive access policies, representation of diverse communities and measures to ensure fair participation. Where relevant, the integration of local, traditional and indigenous knowledge systems should be documented, together with mechanisms ensuring respect for cultural values and practices. Cultural inclusiveness should also be reflected in accessible user interfaces, multilingual or language-sensitive documentation where appropriate, inclusive service design and support mechanisms that reduce barriers for users with different technical capacities, institutional backgrounds or geographic locations.

**C4. Ethical soundness**

This tenet focuses on the requirement that the ERIC’s activities are ethically robust and socially responsible. The guidelines provided by ESFRI require evidence of ethical governance frameworks, including codes of conduct, ethical review procedures and mechanisms for handling conflicts of interest. An Advisory Board on ethics is often expected to be functional at the governance level of the ERIC. The ERIC should demonstrate responsible data management, transparency in operations and accountability towards stakeholders, ensuring that its activities do not create adverse impacts or inequities.

**C5. Effective communication**

This tenet ensures that the ERIC communicates its activities, outputs and impacts effectively to relevant audiences. The ESFRI monitoring approach gives a special emphasis on structured communication strategies, dissemination of scientific results and engagement with stakeholders, including policy-makers and the public. Evidence should include communication plans, outreach activities and indicators measuring visibility, engagement and impact, as well as the use of multiple communication channels tailored to different target groups.

Together, all of the above eleven-tenets provide a comprehensive and integrated framework for the management of Research Infrastructures.

## Discussion

The ten-tenet framework, originally developed in the context of environmental management (Elliott 2013), is based on the premise that successful and sustainable management can be achieved if a sufficiently comprehensive set of key dimensions is identified and addressed. In essence, the concept assumes that, by properly defining and managing a finite number of critical tenets, it is possible to capture most of the relevant attributes required for effective system governance, thereby providing a “good enough” representation of the complexity involved. This approach has been applied in different domains, including the definition of the essential functions of biodiversity data infrastructures (Güntsch et al. 2024) and the assessment and management of non-indigenous species in marine ecosystems (Ojaveer et al. 2014). It has been proposed in solving environmental problems due to marine litter and garbage and leading to promoting the circular economy in dealing with waste (Borja and Elliott 2019) which can then be applied to minimising the environmentally-damanging actions of ERICs. Principles-based thinking has also been applied in biodiversity informatics, where Patterson et al. (2008) proposed thirteen principles for a names-based cyberinfrastructure serving biology, highlighting organism names as a near-universal indexing system for biological data objects. Although narrower in scope than ERIC management, this work further supports the value of explicit principles for structuring complex, distributed and data-intensive scientific infrastructures. Building on this conceptual basis, the present study extends the framework to eleven tenets in order to address the specific characteristics of ERICs, most notably their role in creating the conditions that allow user communities to achieve scientific excellence. In doing so, the framework provides a structured and integrative approach for capturing the key attributes required for the effective management of ERICs.

In line with these conceptual foundations, the eleven-tenet framework presented here emphasises that the ERIC management should be assessed not only through internal organisational processes, but also through the outcomes these processes enable in relation to its mission, vision and strategic objectives. However, while the original tenets were developed in the context of business and environmental systems management, their adaptation to ERICs highlights the need to manage not only technical and organisational components, but also complex socio-technical interactions involving users, stakeholders, governance bodies and policy frameworks. The eleven-tenet framework can, therefore, be seen as complementary to strategic-management tools such as PESTLE analysis: while PESTLE helps ERICs scan their external operating environment, the tenets provide a broader management scaffold through which such external drivers can be integrated with scientific, technological, economic, governance, societal and sustainability considerations.

A key distinction in the context of ERICs is that scientific excellence is not an intrinsic property of the infrastructure itself, but rather an outcome generated by the research communities it supports. Consequently, the effectiveness of an ERIC is determined by its capacity to enable, sustain, amplify and evolve this excellence through the provision of high-quality services, data and collaborative research environments. This reinforces the central role of the newly- introduced tenet of enabling scientific excellence, which acts as the focal point around which all other tenets operate.

The eleven tenets should not be interpreted as independent or sequential elements, but rather as an integrated system in which each component influences and supports the others. As such, Elliott et al. (2025) gives the tenets as a series of Venn diagrams indicating a continuum from an approach to management and then technical measures to achieve the desired outcome. Consequently, failure to adequately address any single tenet may compromise the overall effectiveness of the infrastructure, while their combined and balanced implementation increases the likelihood of achieving long-term sustainability, adaptability, and impact. In this sense, the framework reflects the characteristics of complex adaptive systems, such as SES, where feedback mechanisms, continuous learning, and responsiveness to external drivers are essential (Smith et al. (2025)).

In line with the tiered structure proposed in this study, the successful application of the framework requires coordinated integration across all three levels. At the core tier, this entails the continuous alignment of enabling scientific excellence, technological feasibility and economic viability, ensuring that the infrastructure remains responsive to evolving scientific priorities, emerging technologies and changing funding landscapes. At the structural tier, effective vertical integration across governance levels, European, national, regional and institutional, is essential, supported by coherent administrative, legal and political frameworks. Finally, at the contextual tier, horizontal integration across domains, societal, environmental, cultural, ethical and communications, is required to ensure relevance, legitimacy and long-term impact. Together, these dimensions highlight the importance of adaptive management approaches, supported by robust monitoring systems, such as those developed by ESFRI, enabling the infrastructure to respond dynamically to internal and external drivers.

Ultimately, the eleven-tenet framework provides a structured, yet flexible, approach to understanding and guiding the management of ERICs and RIs in general. It allows for the identification of strengths and gaps, supports strategic prioritisation and facilitates communication with stakeholders, including funders and policy-makers. At the same time, it raises a set of critical questions that must be addressed in order to assess whether an ERIC is being managed successfully. These questions relate, for example, to the extent to which the infrastructure effectively enables scientific excellence; whether it maintains a balance between technological innovation and operational stability; how it ensures long-term financial sustainability; and how it demonstrates its value and impact to society and policy. Addressing these questions requires the development of measurable performance indicators, robust evaluation methodologies and a shared understanding of success criteria across the RI landscape.

The eleven-tenet framework also complements earlier principle-based approaches to ERIC quality and sustainability. The four “golden principles” proposed by LERU (Väänänen and van Tienderen 2017) emphasise access, FAIR data, transparency of information, stable organisational embedding and evidence of operational and scientific quality. These elements are strongly reflected across the eleven tenets proposed here, particularly in relation to enabling scientific excellence, technological feasibility, economic viability, administrative achievement, societal desirability and effective communication. The present framework extends this logic by situating such principles within a broader management structure that also includes political alignment, legal governance, environmental and energy sustainability, ethical soundness and cultural inclusiveness. In this sense, the eleven-tenet framework can be seen as a more comprehensive conceptual scaffold within which existing RI principles, checklists and monitoring approaches can be organised and further implemented.

This convergence is also visible in recent work on open by design research infrastructures (*sensu* Spinello et al. (2021)). Haak et al. (2025) frame RIs as systems that should serve and enrich their user communities, rather than as entities that generate knowledge independently. This is consistent with the central premise of the enabling scientific excellence tenet proposed here: the ERIC does not itself produce scientific excellence, but creates the conditions through which research communities can generate it. Their evaluation framework, which combines adoption, sustainability, governance, support, engagement and trust-building, also resonates with the structural and contextual tiers of the eleven-tenet framework. The latter, therefore, provides a complementary management-orientated scaffold for established ERICs, while open by design offers a useful design-orientated perspective for embedding openness and community value from the earliest stages of infrastructure development.

In this context, the present work lays the conceptual foundation for a subsequent analysis focusing on the practical assessment of ERIC performance. Table 2 already provides an initial evidence-based framework for this purpose, by identifying, for each of the eleven tenets, the type of evidence that would indicate successful and sustainable management of a ERIC. These evidence categories can be reformulated into a corresponding set of key assessment questions, addressing, for example, whether the ERIC enables scientific excellence, maintains adequate technological capacity, secures long-term financial sustainability, operates through effective governance, aligns with policy priorities, responds to societal needs, complies with legal and ethical requirements and communicates its value effectively. The next step will be to implement these questions into measurable indicators, assessment criteria and evaluation methodologies, forming the basis of a subsequent publication.

Finally, another issue concerns the practical impediments to achieving all eleven tenets simultaneously. Some dimensions, such as technological feasibility, economic viability, administrative achievability, environmental sustainability and communication effectiveness, can often be supported by relatively explicit indicators, for example, service availability, budget stability, staffing capacity, energy use, user uptake or outreach metrics. Other dimensions, including political alignment, societal desirability, ethical soundness, cultural inclusiveness and the enabling of scientific excellence, are more difficult to quantify because they depend on context, stakeholder interpretation, long-term effects and qualitative forms of legitimacy. This creates a risk that more easily measurable tenets receive disproportionate attention, while less quantifiable, but equally important dimensions are under-represented in assessment and management. For this reason, the eleven-tenet framework should not be implemented only through numerical indicators, but through a mixed approach combining quantitative metrics, qualitative evidence, stakeholder judgement, case studies and periodic reflective review.

## Conclusions

- ERICs are complex socio-technical systems whose long-term success depends on the balanced management of scientific, technological, economic, administrative, political, legal, societal, environmental, ethical, cultural and communication dimensions.

- The eleven-tenet framework offers an integrated scaffold for organising these dimensions and for translating dispersed management requirements into a coherent structure for ERIC reflection, assessment and development.

- The addition of “enabling scientific excellence” recognises that scientific excellence is produced by user communities, while ERICs provide the data, services, tools, environments and support that make such excellence possible.

- The tenets should be understood as interdependent rather than isolated criteria: weakness in one dimension may affect the resilience, legitimacy, adaptability and sustainability of the whole infrastructure.

- Although grounded in the European RI and ERIC context, the framework addresses management challenges that are relevant to large-scale RIs internationally.

## Figures and Tables

**Figure 1.**
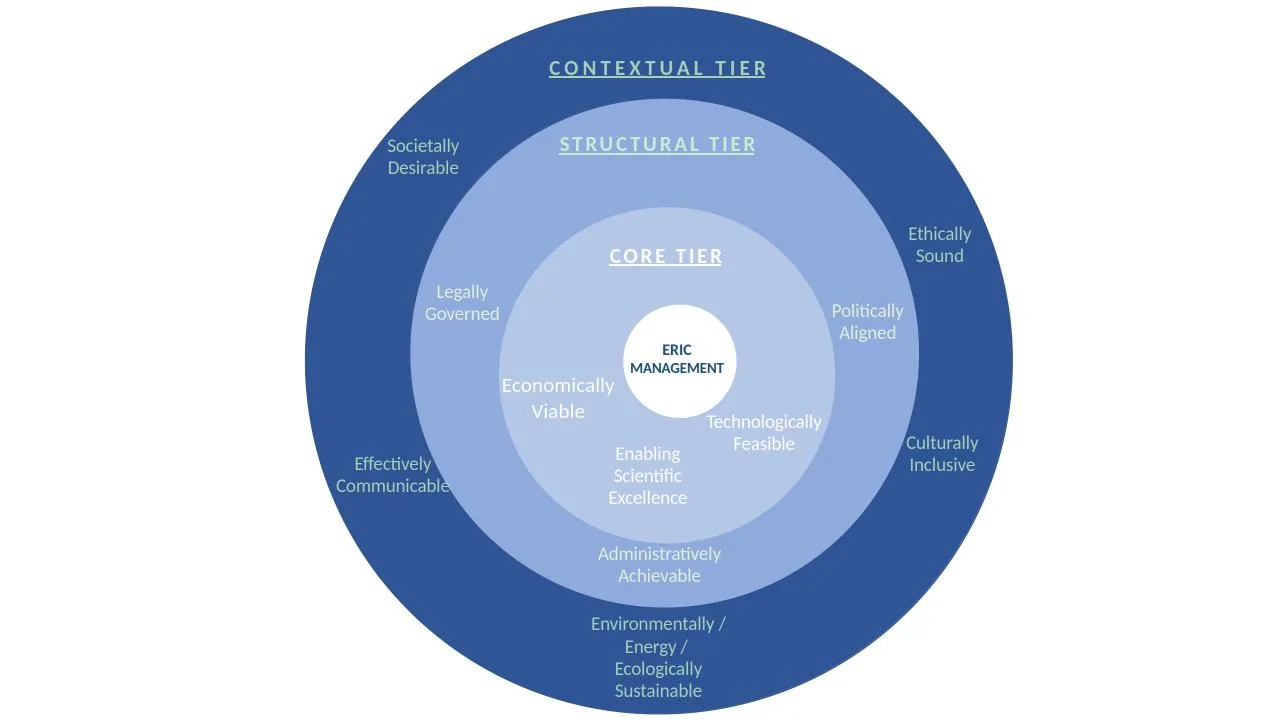
Concentric model of the eleven-tenet framework, illustrating the core, structural and contextual dimensions that jointly support integrated, evolving and sustainable management of ERICs (adapted and extended from Elliott (2013)).

**Table 1.** The *eleven-tenets* for integrated, evolving and sustainable management of ERICs (adapted and extended from Elliott (2013)).

To be successful, integrated and sustainable, the management of RIs should be:
**Enabling scientific excellence** **Technologically feasible** **Economically viable** **Administratively achievable** **Politically aligned** **Societally desirable** **Legally governed** **Environmentally / energy / ecologically sustainable** **Ethically sound** **Culturally inclusive** **Effectively communicable**

**Table 2.** Eleven-tenets showing how successful and sustainable management is achieved for an ERIC.

**Tenet**	**Evidence that it is achieved to give successful and sustainable management of the ERICs**
**Enabling scientific excellence**	The ERIC provides the conditions necessary for research communities to achieve scientific excellence through the provision of high-quality FAIR-compliant data, advanced and reproducible analytical workflows, robust computational environments and effective support to diverse user communities. Evidence includes strong and growing user uptake, high-quality scientific outputs enabled by the ERIC, such as datasets, workflows and publications, support for interdisciplinary research and active engagement of users through co-design and co-creation processes. The ERIC demonstrates continuous adaptation of its services to emerging scientific needs, ensuring its sustained role as an effective enabler of scientific excellence.
**Technologically feasible**	Installations, equipment (both hardware and software) and specialiszed personnel purchased/hired/created and operational; personnel competent in the relevant technologies and to maintain and evolve the technological ecosystem. The RI demonstrates the capacity to evolve with technological developments by integrating new digital tools, computational approaches and service architectures where these strengthen its scientific mission and long-term relevance.
**Economically viable**	Financial aspects secured in the long-term for both the strategic planning and its implementation, preferably by benefitting from a variety of resources from the public and private sectors, including industry; a cost-benefit analysis carried out to show this.
**Administratively achievable**	Processes, procedures, mechanisms and trained and dedicated personnel in operations, adequate resourcing and relevant KPIs for its scientific, technical, financial and administrative performance and evaluation mechanisms at regular intervals, established and SMART targets set and achieved.
**Politically aligned**	Vision, mission, core business and value proposition clearly stated and aligned with the major leading local, national, European and global policy and political demands in the domain of the ERIC; relates to administrative and institutional bodies at local, national, European, regional and global levels; its outcomes fit with national and EU Strategies, Policies and Plans; to ensure the ERIC influences and takes account of the prevailing political discourses.
**Societally desirable**	Users of the ERIC and relevant stakeholders participate in its development, preferably through well-established co-design and co-development processes. The ERIC also addresses major societal challenges, socially tolerated practices and ethically relevant concerns. It promotes equitable communities by ensuring that all groups of users and stakeholders receive the support they need to participate fairly in its governance, management and activities.
**Legally governed**	Ideally, having developed Rules of Procedures, Implementing Rules, Strategies and Policies, which allow its operations under the legal national and EU framework and avoid legal non-compliance and associated risks; actions fit within agreements (soft law) such as for regional seas agreements, European (e.g. Green Deal, Biodiversity Strategy 2030) and at global level (e.g. CBD).
**Environmentally / energy / ecologically sustainable**	The ERIC has an impact on or/and sustainably contributes to the ecological and environmental challenges and has developed an effective Environmental/ Energy Policy, which is in operation and in line with the Green Deal and, for ERICs of the environmental domain, with the Biodiversity Strategy 2030, the Biodiversity Law and relevant Directives.
**Ethically sound**	No conflicts at present or no future adverse legacy or liability, ensuring that costs of the management, maintenance and further development of the ERIC are proportionately placed on its current funders; promote just communities: the systemic barriers are removed, by management, addressing the causes of inequity through justice.
**Culturally inclusive**	Ideally, with clearly stated Cultural Principles and with its Implementation Plan and Business Plan based on these Principles. In addition, all types of user groups, cultural groups and peoples and relevant stakeholders are satisfied, especially indigenous and traditional communities, procedures developed for equity and justice, including gender plan, work-life balance and code of conduct and for the inclusion of the local communities and indigenous peoples.
**Effectively communicable**	Communication and Dissemination Policy developed and in operation, directed to targeted audiences and assessed through defined indicators (KPIs); dissemination through all available channels including both social media and more formal media.
